# Zebrafish Model Insights into Mediterranean Diet Liquids: Olive Oil and Wine

**DOI:** 10.3390/antiox12101843

**Published:** 2023-10-10

**Authors:** Paula Silva, María Rodríguez-Pérez, Emma Burgos-Ramos

**Affiliations:** 1 Laboratory of Histology and Embryology, Department of Microscopy, School of Medicine and Biomedical Sciences (ICBAS), University of Porto (U.Porto), Rua Jorge Viterbo Ferreira 228, 4050-313 Porto, Portugal; 2 iNOVA Media Lab, ICNOVA-NOVA Institute of Communication, NOVA School of Social Sciences and Humanities, Universidade NOVA de Lisboa, 1069-061 Lisbon, Portugal; 3 Biochemistry Area, Faculty of Environmental Sciences and Biochemistry, University of Castilla-La Mancha, Avenue Carlos III s/n, 45071 Toledo, Spain; maria.rodriguezperez@uclm.es

**Keywords:** zebrafish, olive oil, wine, cardiovascular disease, antioxidants, Mediterranean diet

## Abstract

In this review, we explored the potential of a zebrafish model to investigate the antioxidant effects of key components of the Mediterranean diet, namely, olive oil and wine, in the context of preventing age-related diseases, particularly cardiovascular conditions. This paper explores the spectrum of observational studies to preclinical investigations and ultimately converges toward potential translational insights derived from animal experimentation. This review highlights the potential and underutilization of zebrafish as an experimental model in this domain. We highlighted the genetic proximity of zebrafish to humans, offering a unique opportunity for translational insights into the health benefits of olive oil and wine. Indeed, we wanted to focus on the potential of zebrafish to elucidate the health benefits of olive oil and wine while calling for continued exploration to unlock its full potential to advance our knowledge of age-related disease prevention within the Mediterranean diet framework.

## 1. Review Outline

In recent decades, significant attention has been paid to diet, both in scientific and in popular media. A cornerstone of any healthy lifestyle program is the inclusion of a balanced diet that can prevent various diseases, including cardiovascular conditions. Despite the availability of a multitude of diets, only a few have garnered the attention of researchers and have been proven to have health-promoting effects through rigorous scientific studies published in the literature. One of the most notable examples is the Mediterranean diet (MD). The MD has become one of the most studied and widely reported diets and has received a lot of attention. In this literature review, we considered the definition of the MD and its main health benefits regarding longevity and cardiovascular diseases. We provide a brief overview of the antioxidant properties of the MD, especially those of olive oil and wine. We then focus on the potential of zebrafish to elucidate the health benefits of olive oil and wine, calling for continued exploration to unlock its full potential to advance our knowledge of age-related disease prevention within the MD framework. We used PubMed and Scopus as search tools with which to identify articles relevant to our narrative review. Our search was limited to articles written in English, and no date restrictions were imposed. The papers included in this review were not intended to be exhaustive, and in cases where multiple studies were available, we chose the most significant ones.

## 2. The Mediterranean Diet

### 2.1. Mediterranean Diet Definition

The MD is a dietary plan based on Crete’s traditional eating habits. The term “Mediterranean Diet” was coined by Ancel Keys, an American physiologist, in 1960, during the publication of his book *How to Eat Well and Stay Well* [1]. The scientific evidence for its existence before 1960 is controversial, but it is reasonable to consider a broader temporal context, reaching back to biblical times or even earlier [2]. Interestingly, the strength of the MD is closely linked to ancient biblical culture. In fact, when we look for the seven key components of the traditional biblical diet, namely, wheat, barley, grapes, figs, pomegranates, olives, and honey, we recognize some MD components [2]. Olive oil and wine, the foods discussed in this review, were also included in biblical diets. The evolution of the MD is intertwined with the development of Western civilization. Initially, people in the region adapted their food consumption to the seasons, which was determined by climate and agriculture [3]. 

The origins of the MD reflect the cultural, societal, and economic growth of the region. The Latin term “*Mediterranean*” means “the sea in the middle of the earth”, highlighting its historical role as a meeting point between southern Europe, northern Africa, and western Asia. The word “diet” itself is derived from the Greek word “*diaeta*”, which encompasses not only food but also one’s overall lifestyle. The Mediterranean region spans numerous countries, including Italy, Greece, Spain, southern France, Turkey, parts of North Africa, and the Middle East, resulting in a rich diversity of ingredients and culinary traditions within the MD. 

Although the MD has often been associated with the “eternal trinity” of wheat, olive oil, and wine, it also embodies the essence of traditional agricultural practices and dietary habits, which are marked by a culture of sharing and reciprocity [4]. For instance, wealthy urban Greeks favored a diet rich in vegetables, grains, legumes, olive oil, and wine. Barley and wheat were used for oatmeal and bread, whereas various legumes such as fava beans, chickpeas, lentils, lupines, and peas were either prepared in specific dishes or incorporated into flour for bread and oatmeal [5]. A typical MD is characterized by several key features: substantial consumption of vegetables, fruits, legumes, and grains (including complex carbohydrates and dietary fiber), limited total fat intake (<30%), low saturated fat intake (<10%), emphasis on monounsaturated fats, and moderate alcohol consumption (primarily wine) [6]. Over time, the introduction of new ingredients and influences from regions such as Asia and the Americas, including tomatoes, potatoes, maize, beans, and cane sugar, have resulted in changes in Mediterranean cuisine, expanding its culinary horizons beyond indigenous roots. Throughout different historical eras, cultures, religions, agricultural practices, and economic circumstances, the emphasis on food elements within the MD has varied. Additionally, factors such as climate, economic challenges, and scarcity have played a significant role in shaping this diet rather than relying on intellectual foresight or deliberate dietary planning, which is often the approach adopted in modern times to create popular diets. This might explain why it has been challenging to precisely characterize this dietary regimen, as it exhibits unexpected complexity and variations across different countries and historical periods. In 2010, the MD gained recognition as an Intangible Cultural Heritage by the United Nations Educational, Scientific, and Cultural Organization (UNESCO). The official submission made to UNESCO characterizes the MD as “a social tradition rooted in a collection of competencies, wisdom, customs, and traditions that encompass everything from the natural environment to culinary practices. These encompass activities such as cultivation, harvesting, fishing, preservation, processing, cooking, and, most notably, consumption, particularly within the Mediterranean region” [7]. In 2011, the Mediterranean Diet Foundation in Spain, in collaboration with experts in the field, revised the “classic” MD pyramid to accommodate changes brought about by modernization and to integrate cultural and lifestyle aspects [8]. A literature review by Davis et al. (2015) attempted to establish an integrated definition of the MD. In conducting their analysis, the authors considered a variety of criteria, including general descriptive terms, recommended serving sizes of key food groups, and nutrient content [9]. Based on this comprehensive review, the authors defined daily dietary intake as follows: vegetables, 3–9 servings; fruits, 0.5–2 servings; cereals, 1–13 servings; and olive oil, up to 8 servings. In terms of energy content and macronutrient composition, the MD typically consists of approximately 2220 kcal/day, with fat accounting for 37% of the total calories [9]. Regardless of the definition adopted, there is a consensus regarding the health benefits of the MD. Since Ancel Keys’ pioneering research revealed that the dietary practices of Mediterranean countries are associated with longer lifespans and reduced incidences of coronary heart disease (CHD), many more studies have been published [1]. In the following section, we present a summary of the effects of the MD on longevity and cardiovascular diseases (CVDs) derived from observational studies. In our opinion, reliable information regarding dietary effects in humans, especially in those suffering from a specific illness or disorder, requires clinical investigation based on well-constructed preclinical data relevant to the intended human population. Moreover, modern research requires animal studies, such as the analysis of signaling pathways under various conditions or examination of novel dietary approaches. Basic research can benefit from combining animal and human data. Therefore, it is necessary to work with animal models that share genetic similarities. This helps confirm the changes observed in animals after specific treatments in humans. Thus, translational research using observational studies allows for the identification of associations between basic research and humans, even before new experimental strategies are approved and tested in randomized controlled trials (RCTs).

### 2.2. Mediterranean Diet Health Benefits

#### 2.2.1. Mediterranean Diet and Longevity

After the Seven Countries Study, which strongly suggested that low levels of mortality from CHD are strongly associated with the MD [10], scientists began to study the effects of the MD on aging. Aging is defined as a progressive decline in physiological function over time [11]. This natural process is associated with a higher risk of various chronic diseases such as cognitive decline, neurodegenerative diseases, CVD, cancer, diabetes, sarcopenia, and osteoporosis [12]. The aging mechanism is intricate, and nine distinct cellular and molecular characteristics (genomic instability, telomere decline, changes in epigenetic patterns, disruptions in proteostasis, dysregulated nutrient detection, issues with mitochondrial function, cellular senescence, stem cell depletion, and modified intercellular communication) collectively influence the aging trajectory [11,13]. The aging process is not fixed and can be altered by diet and lifestyle factors [13].

It is not surprising that initial studies investigating the impact of the MD on longevity were conducted in Mediterranean countries. Trichopoulou et al. (1995) studied the diets of elderly individuals in three rural Greek villages. They conducted a 5-year follow-up study of 182 elderly Greek participants and showed that those whose diets were different from the traditional MD had a higher risk of all-cause mortality than those who faithfully followed the traditional MD [14]. Trichopoulou also examined how traditional MD affects the survival rate of a large group of elderly men and women aged >60 years at baseline. The results indicated that adherence to the MD was associated with improved longevity in this older population [15]. In Italy, as part of the Italian Longitudinal Study on Aging, 278 participants without dementia were tracked, with an average age of 73.4, for a duration of 8.5 years. Those with a higher intake of monounsaturated fatty acids (MUFA) demonstrated improved longevity. This MUFA-increased intake was significantly associated with a reduction in overall mortality risk [16]. In a Spanish study involving 161 non-smoking men and women aged 65 years, an increase of one unit in the eight-unit MD score was associated with a significant reduction in mortality of 31% among elderly individuals [17]. The “Healthy Aging: A Longitudinal Study in Europe” project was a large-scale cohort study that included healthy men and women aged between 70 and 90 years from 11 European countries. Over the course of a decade, individuals who maintained a healthy lifestyle, characterized by four low-risk behaviors, such as adherence to the medical doctor’s guidelines, moderate alcohol consumption, nonsmoking, and a minimum of 30 min of daily physical activity, have demonstrated a significant reduction in mortality. The lack of adherence to this low-risk pattern was the reason for the 60–64% mortality rate. The authors concluded that pursuing a Mediterranean lifestyle among individuals aged 70–90 years was associated with a 50% decrease in the rates of all-cause and cause-specific mortality [18]. 

The effects of the MD on longevity were examined in regions outside the Mediterranean, where the consumption of monounsaturated fat from olive oil is restricted. In 1988, a European-wide, multicenter study on nutrition and health in the elderly (SENECA: Survey Europe on Nutrition in the Elderly: A Concerted Action) was started to examine dietary patterns in the elderly in relation to lifestyle, social and economic conditions, health, and performance. Nine years later, a paper reporting the results of a study aiming to examine the impact of the MD on life expectancy in a Danish cohort was published. Over a period of six years, a dietary assessment based on seven MD characteristics revealed that a one-unit increase in diet score was associated with a remarkable 21% reduction in mortality [19]. Another important study was the European Prospective Investigation into Cancer and Nutrition (EPIC), which involved 74,607 participants from Denmark, France, Germany, Greece, Italy, the Netherlands, Spain, Sweden, and the UK. The researchers used a modified MD score to assess the degree of MD adhesion among individuals in Europe. In such cases, the monounsaturated fats were replaced with a combination of monounsaturated and polyunsaturated fats to calculate the lipid ratio. Among individuals aged ≥60 years, a higher modified MD score was associated with a lower overall mortality rate. Specifically, a two-unit increase in the score was associated with a statistically significant 8% reduction (with a confidence interval of 3% to 12%). Although this association was more pronounced in Greece and Spain, there was no significant variation in the impact of the score on the overall mortality among different countries. When dietary exposure was adjusted across countries, the reduction in mortality was 7% (with a confidence interval of 1–12%) [20]. In a study involving 818 individuals aged ≥70 years from different European nations, the MD was found to play a significant role in promoting longevity. It did so independently and in conjunction with other factors, displaying a noteworthy impact on survival that was equal to or greater than the influence of all other factors examined in the study [21]. The NIH-AARP Diet and Health Study provided convincing evidence that, among older Americans, following a Mediterranean-style diet regimen significantly reduced all-cause and cause-specific mortality [22]. Reedy et al. (2014) discovered identical outcomes in a study that examined the correlations between four indices, namely, the Healthy Eating Index–2010, the Alternative Healthy Eating Index–2010, the alternate Mediterranean Diet, and Dietary Approaches to Stop Hypertension, and mortality rates from all causes, CVD, and cancer. A decreased risk of all-cause, CVD, and cancer mortality was associated with higher index scores [23].

The MD may also have a positive effect on longevity in younger individuals. Data from Swedish research have shown that the MD can reduce mortality in young individuals. An inverse association was found between closer adherence to the MD, reduced cancer deaths, and decreased all-cause mortality in a cohort of 42,237 young women (aged 30–49 years). There was a 13% reduction in all-cause mortality and a 16% reduction in cancer-related deaths associated with a 2-point increase in diet score [24]. 

A meta-analysis of 12 studies found that over 1.5 million people followed the MD over a period of 3–18 years. A scoring system was set up to determine how closely participants followed the MD. The findings showed that adherence to the MD reduced the likelihood of premature death from any cause by 9%, lowered cardiovascular mortality by 9%, decreased cancer-related mortality by 6%, and diminished the risk of Alzheimer’s and Parkinson’s disease by 13% [25]. A meta-analysis of observational studies, encompassing twenty-nine prospective studies with 1,676,901 participants, found a 10% reduction in all-cause mortality for every 2-point increase in adherence to the MD. The inverse association was stronger in participants residing in the Mediterranean region than in those residing in non-Mediterranean areas according to the subgroup analyses. A nonlinear dose–response analysis also revealed a linear decrease in the risk of all-cause mortality with a greater commitment to the MD [26]. Telomere length, a widely accepted biomarker of the aging process, is longer in those who closely adhere to the MD, as shown in a recent meta-analysis. An analysis of eight original cross-sectional studies and 13,733 participants from five countries found a positive association between adherence to the MD and telomere length [27]. When the MD was followed in 4676 healthy middle-aged women, longer telomeres were observed [28]. 

Observational studies have successfully demonstrated a correlation between adherence to the MD and a reduced risk of overall mortality. However, as assessed by Guasch-Ferré and Willett in 2021, RCTs do not seem to show a substantial effect of the MD on total mortality [29]. Preclinical animal studies may play a pivotal role in bridging this discrepancy in the results. Studies on animals can provide a controlled environment for investigating the precise impact of the MD components on various health outcomes, including longevity. Through animal experiments, researchers can isolate and manipulate specific dietary factors, and closely monitor their effects on mortality. This controlled approach allows for a more nuanced understanding of how the MD influences longevity, potentially shedding light on why RCTs may not consistently reflect the findings of observational studies in human populations.

#### 2.2.2. Mediterranean Diet and Cardiovascular Diseases

Heart and blood vessel disorders are often referred to as CVDs. These include hypertension, CHD, stroke, heart failure (HF), and several other heart-related diseases [30]. CVD is a life-threatening condition and a leading cause of death worldwide [31]. The burden of CVD can be greatly reduced by adopting a healthy lifestyle that includes a healthy dietary pattern [32].

When discussing studies that investigated the association between the MD and CVD, it is mandatory to mention The Seven Countries Study. After a 25-year follow-up period, researchers demonstrated a strong correlation between variations in CHD mortality rates across 16 study cohorts and differences in adherence to the MD within those populations [10]. The EPIC study also played a crucial role in expanding our understanding of how the MD affects CVD. Despite geographic differences, similar outcomes have been observed in various cohorts. In a large prospective survey of 22,043 middle-aged and older adults in Greece, Trichopoulou et al. [15] reported a negative association between adherence to the MD and death due to CHD. An increase of approximately 2/9 in the MD score was associated with a 25% reduction in total mortality and a 33% reduction in mortality due to CHD. These associations were evident regardless of sex, smoking status, education level, body mass index, or physical activity level. It is noteworthy that the correlation between MD scores and mortality was significant among participants aged ≥55 years, whereas it was not observed among younger participants. Increased exposure to a healthier diet, such as the MD, may be the reason for this association [15]. Moreover, in a cohort study conducted on a larger sample of 23,929 apparently healthy women and men in Greece, adherence to the MD was associated with a lower incidence and mortality from CHD [33]. A substantial EPIC cohort of healthy individuals from a Spanish population was followed for >10 years. After adjusting for significant confounders, adherence to a relative MD score was associated with a 40% reduction in the likelihood of a first CHD event [34]. In a Dutch cohort, higher adherence to the MD was associated with a lower risk of a combined CVD endpoint (fatal CVD, nonfatal myocardial infarction (MI), and nonfatal stroke) [35]. 

In addition to the EPIC study, other studies have shown positive effects of the MD on cardiovascular health in non-Mediterranean countries. The MONICA (Multinational MONItoring of Trends and Determinants in CArdiovascular Disease (MONICA)) study examined 1849 Danish men and women and found that each 1-unit increase in adherence to an 8-point scale reduced the risk of CVD by 8% [36]. The Northern Manhattan Study, a cohort study conducted on a large population (*n* = 2568), also revealed that a higher level of adherence to an MD was associated with a decreased risk of MI, stroke, or vascular mortality [37]. 

The ATTICA Epidemiological Cohort Study (2002–2022) included a sample of approximately 3000 individuals aged 18–89 years from the Attica region of Greece. Within the scope of this study, Panagiotakos et al. (2008) [38] explored the association between the MD and CVD events. This study evaluated the 5-year incidence of CVD in a population-based sample of men and women. In both men and women, the 5-year incidence of CVD was 11.0%, and the case fatality rate was 1.6%. Panagiotakos et al. 2008, observed that greater adherence to the MD was associated with a lower 5-year CVD incidence, especially among middle-aged people [38]. Similar findings were obtained in a prospective cohort study involving university graduates from all regions of Spain, in which an inverse correlation was observed between adherence to the MD and the incidence of fatal and non-fatal CVD in initially healthy middle-aged adults. Interestingly, only vegetables, olive oil, and alcohol were significantly associated with CHD risk in a Spanish study, although the inverse association between the MD score and CHD was highly significant [39].

Several observational studies have examined the effects of the MD on women. In a prospective study involving 64,000 postmenopausal women, a higher level of adherence to the MD was associated with a reduction in CVD mortality risk of 18–26% [40]. Furthermore, the MD pattern may be associated with a lower likelihood of sudden cardiac death in women [41]. Recently, a prospective cohort study conducted over 10 years involving 32,921 Swedish women revealed that adherence to the MD was associated with a lower likelihood of MI, HF, and ischemic stroke [42].

In a four-year observational study, individuals from the Health Professionals Follow-up Study and the Nurses’ Health Study were found to be significantly less likely to develop CVD if they improved their adherence to the MD quality scores over time. In the long-term follow-up, the increase in CVD with reduction in diet quality was more pronounced. These findings provide further evidence that modest enhancements in diet quality over time confer benefits for CVD prevention [43].

Researchers have also examined the effects of the MD in patients with a clinical cardiovascular history. Scientists from the CARDIO2000 study examined 661 middle-aged individuals from various regions in Greece who had experienced their first MI or unstable angina episode, along with 661 controls of the same age and sex. The results revealed that adopting an MD resulted in a 7–10% reduction in coronary risk among individuals with hypertension, regardless of whether they were treated, untreated, or had uncontrolled hypertension [44]. Furthermore, in individuals with high cholesterol levels, CARDIO2000 investigators observed that following an MD resulted in a 12% decrease in coronary risk independent of cholesterol levels and other cardiovascular variables [45]. The same researchers also reported that following this dietary regimen resulted in a 35% decrease in coronary risk within a subgroup of individuals with metabolic syndrome, after accounting for sex, education, financial status, and conventional cardiovascular risk factors [46]. The ATTICA study uncovered data from 1188 individuals, unaffected by CVD but exhibiting defined high blood pressure levels (prehypertension) at the time of baseline examination (2001–2002). The five-year follow-up of the study was conducted in 2006, and 798 participants with prehypertension were enrolled. The findings revealed that a substantial proportion of individuals with prehypertension progressed to hypertension over a 5-year period. A multi-adjusted analysis revealed that low adherence to the MD was one of the elements of the profile of prehypertensive individuals who were prone to developing hypertension within a 5-year period [47]. Furthermore, the MD decreased the 10-year CVD risk among smokers and sedentary and obese subjects. Adherence to the MD resulted in a significant reduction in CVD risk irrespective of various factors. Therefore, even subjects with unhealthy lifestyle behaviors may benefit from adherence to this diet, suggesting another dimension of prevention strategy [48].

Several case-control studies have contributed to the growing body of evidence supporting the health benefits of the MD. In a case-control study conducted in Greece (CARDIO2000), investigators discovered protective associations for the primary prevention of acute coronary syndrome. Each 10-unit increase in the MD score was associated with 27% lower odds of acute coronary syndrome (95% CI: 0.66–0.89) [49]. Applying the same score to a Spanish case-control study involving 171 patients and 171 matched controls, researchers discovered that the probability of sustaining MI decreased as the MD score increased. Moreover, even after adjusting for primary cardiovascular risk factors, a significant linear correlation was observed between the diet score and the risk of MI. These findings support the idea that the MD can effectively reduce the risk of MI [50]. In a hospital-based case-control study, researchers assessed adherence to the traditional MD using the MD scores. This score was based on nine dietary components, including high consumption of vegetables, legumes, fruits, nuts, cereals, fish, and seafood, as well as a high ratio of monounsaturated to saturated lipids. In contrast, low consumption of dairy and meat, along with moderate alcohol intake, were considered. The association between the MD score and its individual components and the risk of acute MI was investigated using multiple logistic regression models, while controlling for potential confounding variables. This study was conducted in Italy, between 1995 and 2003. This study demonstrated that adherence to the MD was associated with a reduced risk of acute MI [51]. Another case-control study that investigated cardiovascular events highlighted the influence of anxiety and depressive symptoms on cardiovascular events. The participants with low anxiety levels exhibited stronger adherence to the MD, which emerged as a significant protective factor. The study included 1000 individuals from Greece, half of whom had previously experienced an episode of acute coronary syndrome or stroke. Therefore, the authors propose including assessments for anxiety and depressive symptoms as part of the baseline evaluation for primary cardiovascular prevention in apparently healthy individuals. To achieve synergistic effects, they recommended combining dietary interventions with psychological treatments [52]. These results were consistent with those of another cohort study of 30,000 participants in the United States, where high adherence to the MD was associated with a lower risk of incident stroke, independent of potential confounders [53].

The PREDIMED (Prevention with Mediterranean Diet) study was designed to assess the long-term effects of the MD on incident CVD in men and women at high cardiovascular risk in Spain from 2003 to 2011. They recruited 7216 participants, aged 55–80 years, who had no CVD at enrollment but were at high cardiovascular risk because of the presence of type 2 diabetes or at least three of the following risk factors: current smokers, hypertension, high low-density lipoprotein (LDL)-cholesterol, low high-density lipoprotein (HDL)-cholesterol, overweight or obesity, and family history of premature CVD. The median follow-up was 4.8 years. For the first time, the findings demonstrated that an MD supplemented with either extra virgin olive oil (EVOO) or nuts reduced the risk of developing CVD by 30% and by 50% in the case of peripheral arterial disease [54].

While observational studies have shed light on the cardiovascular benefits of adhering to the MD, there are several critical questions that require further investigation. These inquiries extend beyond the epidemiological context and explore into the specifics of the diet itself, including the relative impact of individual food components, and the need to eliminate or replace items to enhance its benefits. Addressing these questions necessitates a focused approach involving the investigation of individual food components, formulation of informed dietary recommendations, and conduct of thorough animal trials. Furthermore, a profound connection between the MD and gut health emerges, highlighting the importance of maintaining a harmonious gut, characterized by diverse microbial profiles and regular bowel habits, in promoting overall physical well-being and serving as a shield against chronic diseases including CVDs. Several factors, including dietary changes and stress, can disrupt this balance. The MD plays a significant role in restoring and maintaining this equilibrium. Although the current understanding is promising, it is important to have caution when extrapolating these findings to human health and to continue further research in this area [55].

## 3. Translational Research: From Observational to Animal Studies

### 3.1. One Type of Effect at a Time: Here, the Antioxidant

Foods are macro- and micronutrients that are necessary to maintaining organism growth, vital processes, and energy supply. Furthermore, some nutrients and other molecules present in food possess antioxidant properties, which are beneficial to human health. The term antioxidant is defined as an organic or inorganic chemical compound that inhibits the oxidation of molecules and protects cells and organisms from oxidative damage caused by free radicals. These free radicals are highly reactive molecules that contain unpaired electrons and can damage cells, proteins, DNA, and other biomolecules by stealing electrons, leading to cellular damage and aging. High production of peroxynitrile and hydroxyl radicals also peroxides lipids, altering the cellular membrane and the structure of lipoproteins [56].

During food processing, lipids deteriorate via oxidation, thereby generating potentially toxic substances. There are three common mechanisms of lipid oxidation: photo-, auto-, and enzyme-mediated mechanisms. Vegetable oils, which contain high levels of polyunsaturated fatty acids, are very sensitive to lipid oxidation, being the main reason why these kinds of oils have elevated amounts of antioxidants, principally in their phenolic compounds [57,58]. These compounds are normally biosynthesized from tyrosine or phenylalanine, and their antioxidant properties are attributed to the hydroxyl groups on the benzene ring. Phenolic antioxidants are widely studied in diverse plant foods such as fruits, cereals, vegetables, seeds, wine, or vegetable oils, as it has previously said [58]. Antioxidants can be classified, according to their mechanism of action, as primary and secondary. Primary antioxidants neutralize free radicals by donating a hydrogen atom or transferring a single electron, while secondary antioxidants neutralize prooxidant catalysts [58].

Aging is the progressive loss of organ and tissue function due to the passage of time. The oxidative stress theory of aging is based on the structural damage-based hypothesis that age-associated functional losses are caused by the accumulation of oxidative damage to macromolecules (DNA, proteins, and lipids) by reactive oxygen species (ROS). These ROS are essential for diverse physiological functions but can be toxic at high levels [59] because they probably produce cellular senescence, a physiological mechanism that blocks cellular proliferation due to alterations that occur during replication. Mitochondria are the primary endogenous source of ROS production because of their role in adenosine triphosphate production via oxidative phosphorylation when molecular O_2_ is reduced to H_2_O in the electron transport chain. Furthermore, it is well established that mitochondrial superoxide is an important source of cellular ROS. ROS produce cardiac myopathy because of mitochondrial damage, and it has been shown that the kidneys and liver are targets of ROS because they are directly related to metabolic and filtration processes. In the kidneys, ROS initiate the synthesis of different proinflammatory cytokines, ultimately affecting renal function; in the liver, they alter hepatocyte membranes, leading to fibrosis and cirrhosis [56]. Recent studies have also shown that oxidative stress epigenetically alters chromatin, changing the way in which the genes are expressed. These variations may initiate physiological responses to oxidative stress, thereby facilitating the development of diverse illnesses [59].

Having said that, and although age-related diseases are multifaceted and involve multiple factors and pathways, studying a single metabolic pathway in isolation, such as oxidative stress, remains a critical and valuable approach. This method offers clarity, precision, and potential for targeted interventions, providing a foundational understanding that can enhance our ability to manage and treat complex diseases. The critical role of oxidative stress in aging and CVDs underscores the importance of adopting an MD rich in antioxidants.

### 3.2. One Food at a Time: Here, the Liquid Foods

In the MD, there are two outstanding liquid foods: olive oil and wine. Ancient civilizations were great consumers of olive oil not only in their daily diets but also as a medicine and source of light with which to illuminate their homes during the Middle Ages. All observational data indicated that our ancestors observed some beneficial effects of olive oil on health. However, the chemical substances or constituents responsible for these effects were not investigated. Wine has been a popular beverage for millennia, with historical evidence dating back to around ten thousand years. Wine has played a significant role in numerous medicinal applications throughout the history of humankind. Approximately five decades ago, scientific research was initiated to investigate the beneficial effects of moderate alcohol consumption on cardiovascular mortality. With the discovery of the French paradox, there has been a substantial boost in biological studies related to wine [60].

Olive oil is a crucial liquid food for the MD as it is the primary source of fat and offers numerous health benefits. EVOO is especially beneficial as it retains its organoleptic and nutritional properties after extraction and contains high levels of MUFA, tocopherols, and polyphenols. From a chemical point of view, 98–99% of the total weight of EVOO is represented by fatty acids, especially MUFA such as oleic acid. Tocopherols, polyphenols, and other minor constituents represent the remaining 1–2% which have antioxidant properties that contribute to the health effects of EVOO. EVOO has been linked to various preventive and therapeutic health benefits for different pathologies, including cardiovascular and inflammatory diseases, obesity, cancer, and neurodegenerative alterations associated with aging [61,62].

Several observational studies have shown that the minor constituents of olive oil mediate its health benefits, attenuate CVD prevalence, reduce the risk of aging-associated diseases, decrease weight in obese patients, and increase longevity [62,63,64]. Over the last decade, positive feedback from the scientific community has motivated researchers to further investigate the role of the minor constituents of olive oil in the treatment of various global pathologies. Olive oil has been attributed to its antioxidant, anti-inflammatory, antiatherogenic, antithrombotic, antiaging, neuroprotective, and antimutagenic properties, with many yet to be discovered [63,65,66]. Several in vitro studies have demonstrated the beneficial effects of olive oil. However, simple and suitable animal models are required in order to determine their nutraceutical advantages.

Wine is a notable source of polyphenols, some of which have antibacterial, antifungal, antiviral, antineoplastic, and anti-inflammatory properties. Furthermore, it has been reported that their therapeutic use is beneficial in several diseases, including CVDs and other diseases associated with aging. These pharmacological effects are primarily associated with their antioxidant capacity [67]. Owing to its greater content of antioxidant substances released from the grape skin and seeds, red wine is considered to have a more protective effect. In a bottle of red wine, the total polyphenol content is around 1.8 g/L, whereas in a bottle of white wine, it ranges from only 0.2 to 0.3 g/L of polyphenols. During the process of producing white wine, the skin and seeds are promptly removed from the must, which is then left for fermentation. Since in vitro antioxidant capacity is closely linked to total polyphenol content, white wines exhibit approximately five to ten times lower antioxidant activity than red wines. White wine also contains significant amounts of hydroxycinnamic acids, tyrosol, and hydroxytyrosol, which are known for their antioxidant properties [65,68]. One noteworthy aspect of this diversity is the variation in antioxidant content among the different types of wine. The specific antioxidants and their levels can vary depending on the grape variety, terroir, and winemaking technique. In recent years, there has been a growing interest in organic winemaking, including its subsets, such as biodynamic, natural, and clean wine. Organic winemaking emphasizes environmentally friendly practices and avoids the use of synthetic chemicals. This approach benefits not only the environment, but also the antioxidants present in wine [69]. Understanding the intricacies of winemaking methods is crucial. For example, biodynamic winemaking incorporates holistic farming principles, enhancing the overall vitality of the vineyard and potentially leading to unique antioxidants in grapes. Natural wines are crafted with minimal intervention, allowing grapes to express themselves fully and potentially preserving a broader range of antioxidants [69]. Additionally, the rise of clean wine, which focuses on transparency regarding what goes into the bottle, ensures that consumers receive wines free from additives and unnecessary processing, retaining the natural antioxidants present in grapes. In this evolving landscape, wine enthusiasts seek not only exquisite tastes but also health-conscious choices, exploring the diverse world of antioxidants in different types of wine, especially those emerging from organic winemaking and its subsets [69].

As previously mentioned, numerous observational studies have been conducted over the past few decades to investigate the health effects of the MD, including moderate alcohol consumption (mainly wine). Moderate alcohol intake is associated with reduced CVDs risk and overall mortality in both healthy and unhealthy individuals. Observational studies examining the correlation between alcohol consumption and mortality have demonstrated that individuals who refrain from consuming alcohol have a greater likelihood of death and cardiovascular incidents [70,71]. In the ATTICA study, an interesting pattern emerged regarding the relationship between low wine or beer consumption and the risk of developing CVD. When individuals consumed less than one glass per week, a distinct and striking correlation was observed, indicating a decreased risk of CVD for wine (hazard ratio: 0.40, 95% CI: 0.17–0.98) and beer (hazard ratio: 0.43, 95% CI: 0.20–0.93) compared to individuals who did not consume alcohol. However, no significant relationship was observed between abstainers and individuals who consumed more than one glass per week. Nonetheless, compared with subjects who consumed less than two grams of ethanol per day, those who consumed between 2 and 10 g, 10 and 20 g, and more than 20 g of ethanol per day had a higher hazard ratio [72]. The Moli-sani study, a large-scale cohort study, examined the association between alcohol consumption and the onset of HF and ventricular fibrillation. The study revealed that moderate alcohol consumption, ranging from 10 to 20 g/day, was associated with a reduced risk of HF; however, it did not exhibit a comparable effect on AF when compared to individuals who abstained from alcohol. In addition, this study examined certain biochemical parameters, including HDL and total cholesterol, in relation to alcohol consumption. Those who consumed more than 48 g of alcohol per day had the highest HDL levels (63 mg/dL). Nonetheless, their total cholesterol levels were the highest in the entire study cohort, averaging 226 mg/dL. In contrast, non-drinkers had HDL levels of 54 mg/dL, and occasional drinkers had total cholesterol levels of 207 mg/dL. The authors found that the mean reduction in the risk of total mortality associated with alcohol consumption was 11%. Additionally, alcohol consumption of >20 g/day was linked to a 13% reduction in the risk of total mortality. Considering the deaths attributed to cardiovascular causes alone, these trends were consistent. Notably, the correlation between alcohol consumption and mortality was similar between men and women. Furthermore, the observed benefits were more pronounced in individuals who favored wine, suggesting that the observed benefits might be attributed to components other than ethanol [73]. In summary, observational studies have demonstrated a protective effect of moderate alcohol consumption on the occurrence of cardiovascular events; however, the precise mechanisms involved are still not sufficiently understood.

Animal trials to study the antioxidant effects of specific foods such as olive oil and wine offer numerous advantages. It helps to identify active components, provides experimental control, establishes dose–response relationships, offers mechanistic insights, has practical applications, and can be cost-efficient, reducing the expense of sociosanitary treatment of determinate pathologies. These insights can contribute to a deeper understanding of the health benefits associated with the components of the MD and can inform dietary recommendations. Wine and olive oils were selected for studies on the antioxidant effects of the MD because of their prominence in the diet, distinct antioxidant profiles, existing scientific interest, ease of isolation and control, consumer relevance, and documented positive health outcomes. Studying these specific components allows researchers to explore the mechanisms responsible for the potential health benefits of the diet.

#### 3.2.1. Olive Oil and the Animal Studies on Its Antioxidant Properties

Epidemiological studies have demonstrated that olive oil is rich in antioxidants, which can help prevent oxidative stress, which contributes to the development of diseases such as CVD, cancer, and neurodegenerative diseases [57,74,75]. Olive oil phenols are potent antioxidants because of their capacity to scavenge ROS produced by oxidative stress and stimulate the activity and synthesis of antioxidant enzymes. A recent study observed that the activity of the antioxidant enzymes superoxide dismutase (SOD), catalase (CAT), and glutathione peroxidase (GPx) increased in the liver and white fat tissue of control rats fed with olive oil for 30 days [76].

To date, in vivo studies have focused on two approaches: supplementation of the diet with olive oil and analysis of the evolution of biomarkers in a determinate disease, or treatment with one specific polyphenol to analyze its antioxidant capacity, adjust the dose, and describe its mechanism of action. This last approach allows us to attribute beneficial effects, qualitatively and quantitatively, to specific polyphenol components that can be used as nutraceuticals. Concurrent supplementation with olive oil significantly reduced circulating liver function marker enzymes in rats with hepatic injury and exerted a nephroprotective effect in rats with acrylamide-induced renal damage [77,78]. In rats with experimentally induced Alzheimer’s disease, olive oil decreased beta-amyloid peptide and tau protein accumulation in the hippocampus, improving cognitive decline [79]. Likewise, sustained treatment with olive oil reduced blood pressure, cholesterol levels, and atherosclerotic plaque formation in hypertensive rats, exerting a cardioprotective effect [80]. However, we must not forget that all pathologies share a common factor, namely, oxidative stress, which is also targeted by olive oil. The content of antioxidant enzymes (SOD, CAT, and GPx dismutase) was ameliorated in the liver and increased in the kidneys and pancreas of rats with hepatic alterations and in diabetic rats, respectively [77,81]. An olive oil-enriched diet decreased lipid peroxidation and ROS in the hippocampus and increased GPx levels in the prefrontal cortex of rats, thereby improving learning and memory [82]. Numerous studies have suggested that olive oil plays an important protective role against DNA damage initiated by free radicals in animal models of cancer [83].

In summary, animal models of different diseases suggest that a diet supplemented with olive oil exerts many beneficial effects on human health, as many of these antioxidant effects are mainly induced by olive oil polyphenols.

#### 3.2.2. Wine and the Animal Studies on Its Antioxidant Properties

Numerous animal studies have consistently shown that moderate and prolonged wine consumption offers substantial protection against oxidative stress. These effects have been observed in various organs including the liver, kidneys, and brain. Roig et al. (1999) assessed the impact of moderate consumption of red wine on the antioxidant system present in the liver, kidney, and plasma of rats. After 45 days of red wine consumption, there was an increase in liver SOD and GPx activities. The consumption of wine and ethanol led to lower levels of liver malondialdehyde and increased liver CAT activity during both periods. After 45 days of wine consumption, the reduced glutathione/oxidized glutathione ratio was higher in the kidney, whereas malondialdehyde levels were lower after six months of wine consumption. After six months of treatment, malondialdehyde levels were reduced in the plasma [84]. In another study, for 28 days, Sprague Dawley rats were fed four different beverages: red wine, alcohol solution, de-alcoholized wine, and water. There were no significant differences in CAT and GPx levels in the liver, whereas SOD levels were significantly lower in wine-treated animals. The activity of erythrocytic GPx was lower in the group that consumed red wine. In all studies, the authors suggested that the observed effects were attributable to the non-alcoholic components of wine [85]. Wistar rats subjected to induced inflammation and oxidative stress showed reduced concentration of malondialdehyde in the liver following the administration of red wine [86]. Moreover, research has demonstrated that red wine can alleviate oxidative stress in the liver of rats exposed to alcohol, thereby preventing fatty liver and hepatic fibrosis [87].

Ethanol is metabolized to acetaldehyde by liver alcohol dehydrogenase and microsomal cytochrome P450 2E1 (CYP2E1). The administration of red wine, containing 55.2 mg total flavonols, to Wistar rats for 10 weeks resulted in inhibition of alcohol-induced liver CYP2E1 protein expression [88]. Moreover, chronic consumption of alcohol led to an increase in the expression of CYP2E1 and antioxidant proteins in the liver without causing any alcohol-related oxidative stress or alcoholic liver steatosis [89]. Red wine administration also decreases cytochrome P450 activity in rat kidneys [90]. Ethanol metabolism by CYP2E1 results in the production of metabolites that induce ROS production, which intensifies oxidative stress. However, in a study conducted by Rodrigo et al. (2005), no significant differences in thiobarbituric acid reactive substance (TBARS) levels were detected in mature Wistar rats administered either wine or an ethanol solution (containing 12.5% ethanol) for one month. This lack of difference was likely due to the antioxidant properties of the polyphenolic compounds present in wine, which may have protected against LDL oxidation and subsequently prevented oxidative stress [91].

In experiments involving type 1 diabetes, an enriched wine concentrate containing natural polyphenols effectively regulated hyperglycemia levels, normalized hemoglobin concentrations, and affected erythrocyte count. Remarkably, the administration of this wine concentrate inhibited lipid peroxidation and oxidative modifications in plasma proteins in rats with experimental diabetes mellitus. This results in increased SOD activity and decreased CAT and GPx activities [92]. An in vivo study strongly suggested that white wine, when enriched in polyphenols, can induce ethanol-independent effects in a model of insulin-deficient diabetes characterized by significant oxidative stress. These findings indicate that polyphenols can enhance certain mechanisms associated with microangiopathy, an effect that is absent when diabetes is corrected by alcohol [93]. Recently, it was shown that prolonged consumption of red wine may enhance oxidative stress management and the functioning of angiotensin II and 1-adrenoceptors [94]. This provides new avenues for pharmacological and dietary therapeutic strategies in the management of hypertension and diabetes.

A recent study aimed to examine the efficacy of red wine, with a dosage of 7 mL/kg/day, which is equivalent to approximately 16.5 mg/kg/day total polyphenols, in comparison to white wine, with a dosage of 7 mL/kg/day, which is equivalent to approximately 1.7 mg/kg/day total polyphenols, in preventing acrylamide-induced subacute hepatic injury and oxidative stress in Wistar rats. The authors found that regarding hepatic enzyme activities, the administration of red wine significantly reduced aspartate aminotransferase values, whereas for alanine aminotransferase values, only a normalization tendency was observed. Treatment with red and white wine significantly prevented the increase in malondialdehyde and TBARS levels, as well as the depletion of glutathione. Red wine treatment normalized the activities of the antioxidant enzymes CAT and SOD in rats intoxicated with acrylamide, whereas white wine supplementation did not produce significant differences in antioxidant enzyme activities. These findings demonstrate that red wine has a significant protective effect on oxidative stress and liver injury induced by acrylamide in rats through its antioxidative activity, due to its higher phenolic content [95].

The antioxidant properties of red wine safeguard rat hippocampal neurons against ethanol-induced damage by reducing lipid peroxidation, augmenting antioxidant defenses (glutathione antioxidant system) and stimulating antioxidant enzyme activities in rat models, resulting in a boost in spatial learning and memory [96]. The cerebellum was found to be more affected by ethanol consumption than other brain areas because of the lower basal level of antioxidant defenses and higher concentration of oxidized lipids [97].

In summary, red wine intake has been associated with increased antioxidant enzyme activity, reduced lipid peroxidation, and improved oxidative stress management in animals. Red wine has also been shown to be effective in reducing oxidative stress induced by factors such as alcohol consumption, diabetes, and exposure to organic compounds, such as acrylamide. Furthermore, it has been found that red wine can influence the expression of liver enzymes, such as CYP2E1, involved in ethanol metabolism, potentially reducing alcohol-induced oxidative stress. Red wine also demonstrated protective effects on brain neurons against ethanol-induced damage, thereby enhancing cognitive ability. These effects are attributed to the non-alcoholic components of wine, particularly polyphenolic compounds. Notably, enriched wine concentrates and polyphenol-enriched white wine have shown promising results in terms of managing oxidative stress-related conditions, including diabetes and acrylicmide-induced liver injury. Although in vivo studies with rodents have provided valuable insights into the potential benefits of red wine consumption, there are still unanswered questions and limitations. Before conducting human clinical trials, it is important to explore alternative animal models to address these limitations and provide a more comprehensive understanding of how red wine affects oxidative stress and overall health in humans.

### 3.3. Zebrafish as a Potential Model to Illustrate Olive Oil and Wine Antioxidant Effects

**Figure 1:**
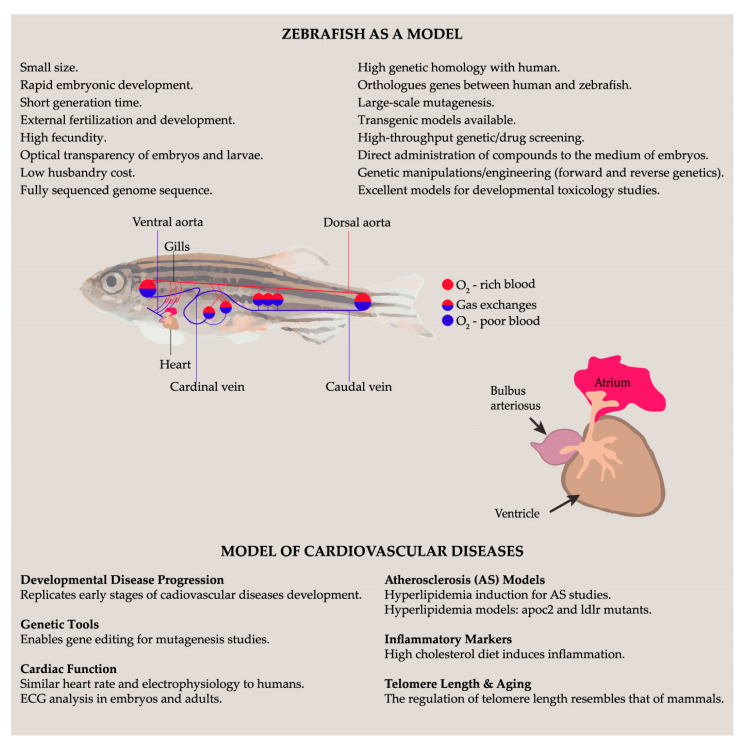
Advantages of zebrafish model and how it can be used to study cardiovascular diseases. Simple representation of zebrafish cardiovascular system. From the heart, O_2_-poor blood goes to the ventral aorta, which branches out, molding the afferent branchial arteries and leading the blood to the gills. Exchanges occur, and the blood becomes rich in oxygen, draining into the efferent branchial arteries and the dorsal aorta. From these vessels, blood travels to the anterior and posterior capillary beds, in which it becomes oxygen-poor again; then, it re-enters the heart through the caudal and cardinal veins. Figure also includes a characterization of the small fish heart, highlighting its four chambers: Sinus venosus, atrium, ventricle, and bulbus arteriosus.

Traditionally, mammalian models, especially rats and mice, have been the primary models used for research in food science, although their complexity and relatively slow rate of development often compromise the rapid progress in resolving fundamental nutritional questions. However, new animal models such as zebrafish and frogs, whose genomes are fully sequenced and very similar to humans, have emerged [98]; 70% of human genes have at least one zebrafish ortholog. Zebrafish have several advantages as model organisms because of their short life cycle, strong reproductive ability, easy rearing, and low cost (Figure 1). Therefore, the use of zebrafish in nutritional studies provides a valuable reference for researchers in the field of food science [99].

Zebrafish are emerging as very high-throughput and genetically tractable vertebrate models for replicating the developmental environment of disease progression in CVD pathophysiology. A diverse range of gene editing tools have been developed to propagate the modified contractile apparatus or CVD pathophysiology in multiple zebrafish generations, thereby enabling the production of multiple, stable zebrafish generations for the purpose of studying mutagenesis [100]. Zebrafish have a great potential for cardiac function disorders. Their heart rate is similar to that of humans compared to other model organisms. Electrocardiogram (ECG) analysis of adult zebrafish underscores the high similarity in electrophysiology between the adult zebrafish heart and that of humans. Although zebrafish hearts are small, techniques have been developed to measure ECG profiles in embryos. These methods have identified similar ECG features in embryos as early as three days post-fertilization (dpf), which mirrors those observed in adult zebrafish (Figure 1) [101]. Atherosclerosis (AS) is a multifaceted chronic disease that poses a significant threat to human health, and it is a fundamental factor underlying numerous CVDs. In clinical practice, one of the most prevalent diagnostic indicators of AS is the presence of abnormal blood lipid levels, which are frequently observed in patients with AS due to disturbances in lipid levels. Typically, cholesterol is transported to peripheral tissues via LDL and subsequently returned to the liver via HDL through its cholesterol reverse-transport function, ultimately leading to its elimination. However, when exposed to oxidative stress, LDL is frequently oxidized into ox-LDL, which is avidly engulfed by macrophages, giving rise to foam cells. This disrupts normal cholesterol metabolism. These foam cells actively contribute to the formation of atherosclerotic plaques and rupture can precipitate ischemic heart disease or stroke. Ox-LDL also has detrimental effects on AS by damaging the vascular endothelium, stimulating the migration and proliferation of smooth muscle cells, and activating platelets. Consequently, inhibition of LDL oxidation has emerged as a promising avenue for enhancing lipid metabolism, preventing the onset of AS (Figure 1) [102]. Hyperlipidemia can be effectively induced in zebrafish through both dietary and genetic strategies, thus providing valuable models for screening novel lipid-lowering compounds. Currently, there are two established zebrafish genetic models of hyperlipidemia: apolipoprotein C-II (apoc2) and LDL receptor (ldlr) mutants [103,104]. In accordance with the clinical phenotype observed in human patients with APOC2 deficiency, zebrafish mutants deficient in Apoc2 exhibit hypertriglyceridemia when fed a standard diet. Apoc2-deficient zebrafish larvae exhibit lipid accumulation and lipid-laden macrophages within their vasculature, similar to the early atherosclerotic lesions observed in humans and mice [103]. In contrast, ldlr mutants develop moderate hypercholesterolemia when fed a standard diet. This hypercholesterolemic state is further aggravated following a short-term, 5-day high cholesterol diet (HCD) regimen initiated 4.5 days post-fertilization (dpf) (Figure 1) [104]. Feeding zebrafish a high-cholesterol diet (HCD) closely mimics certain processes observed in the early AS stages, including hypercholesterolemia, lipoprotein oxidation, vascular lipid accumulation, and recruitment of myeloid cells to the vasculature [105]. It was observed that feeding zebrafish an HCD for up to 10 days, beginning at 5 dpf, resulted in increased expression of inflammatory markers, such as tumor necrosis factor-alpha (TNF-α) and interleukin-1 beta (IL-1β). Additionally, there is a reduction in the expression of the gene encoding the anti-inflammatory protein peroxisome proliferator-activated receptor-γ in the endothelium before myeloid cell accumulation and lipid deposition occur [106]. Numerous studies have demonstrated that telomeres are shorter in human arterial tissue located in vascular beds that are susceptible to AS than in those located in sites that are resistant to AS. Furthermore, the telomere length of white blood cells is shorter in individuals with conditions such as hypertension, diabetes, and coronary artery disease than in unaffected controls (Figure 1) [107]. The regulation of telomere length in zebrafish resembles that in mammalian organisms, making it an ideal model for investigating the link between telomeres and vascular aging (Figure 1) [108]. In conclusion, the zebrafish model has emerged as a valuable tool for advancing our understanding of CVDs, particularly AS and hyperlipidemia. This versatile model combines the practical advantages of in vitro systems and the physiological relevance of mammalian models. Zebrafish share a substantial degree of genetic and functional similarity with humans, making them an ideal platform for investigating the intricate mechanisms underlying these diseases.

**Figure 2:**
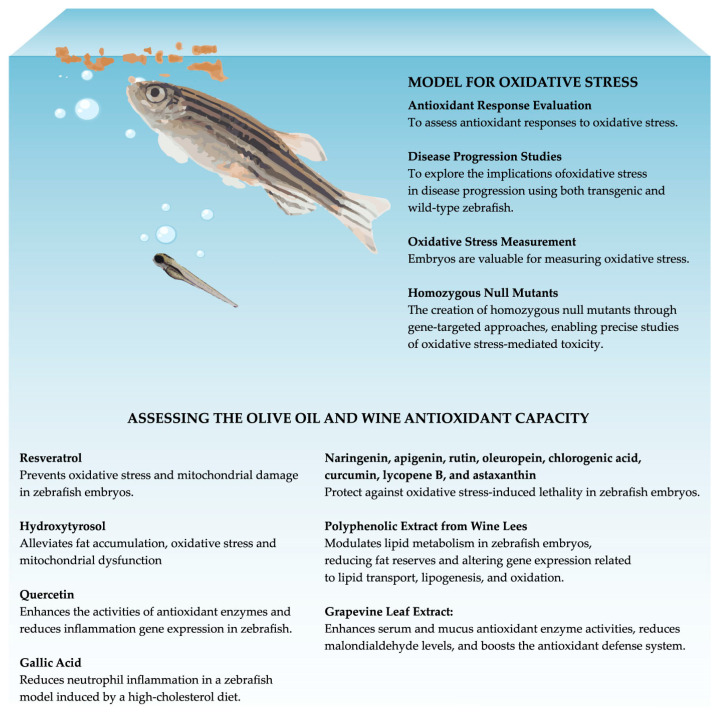
Zebrafish model to assess the olive oil and wine antioxidant capacity.

Zebrafish present an exceptionally promising model organism, exhibiting significant potential for unraveling the mechanisms associated with ROS in crucial human diseases. Studies have demonstrated their ability to evaluate antioxidant responses to oxidative stress in vivo, thus making them a rapid and straightforward experimental model. Researchers have explored the effects of oxidative stress on disease progression in both transgenic and wild-type zebrafish. Remarkably, zebrafish embryos are invaluable for conducting in vivo experiments and devising protocols for measuring the oxidative stress in living organisms. Recent advancements in zebrafish research include the generation of homozygous null mutants using a gene-targeted approach. This innovation allows for precise study of oxidative stress-mediated toxicity [107]. Zebrafish are also used as a model with which to evaluate the antioxidant efficacy of polyphenols, including those found in olive oil and wine. Resveratrol (5 µM), an important wine polyphenol, was shown to prevent oxidative stress and mitochondrial damage in zebrafish embryos (Figure 2) [109]. Administration of quercetin (1 µg/L) significantly enhanced the activities of SOD, GPX, and CAT, as well as the expression of the respective genes. Moreover, the expression of inflammatory genes in the liver and intestine of zebrafish is reduced (Figure 2) [110]. In addition, a recent study showed that zebrafish fed a diet supplemented with 200 mg/kg of hydroxytyrosol, the main polyphenol present in EVOO, alleviates fat accumulation, oxidative stress, and mitochondrial dysfunction (Figure 2) [111]. In a previous study, quercetin (50 and 100 mg/kg; administered intravenously) was found to inhibit lipopolysaccharide-induced behavioral changes, neuronal disruption, and levels of TNF-α, IL-1β, lipid peroxidation, and acetylcholinesterase in adult zebrafish (Figure 2) [112]. Administration of gallic acid resulted in a reduction in the neutrophil inflammation index in a zebrafish inflammatory model induced by a high-cholesterol diet (Figure 2) [113]. Arteaga et al. (2021) evaluated the in vivo protective effects of six phenolic compounds (naringenin, apigenin, rutin, oleuropein, chlorogenic acid, and curcumin) and three carotenoids (lycopene B, β-carotene, and astaxanthin) that are naturally present in foods using a zebrafish embryo model. They found that each compound, except β-carotene, had a protective effect against oxidative stress-induced lethality (Figure 2) [114].

A polyphenolic extract derived from wine lees has a noteworthy impact on the regulation of lipids in zebrafish metabolism. The impact of this extract on lipid metabolism in zebrafish embryos resulted in a decrease in fat reserves and modifications in the expression of crucial genes associated with lipid transport, lipogenesis, and oxidation. Alterations in the concentrations of stearic and oleic acids, as well as polyunsaturated fatty acids and total fatty acids, within the phospholipid and triglyceride fractions of zebrafish embryos, provided evidence of the remodeling and antioxidant capabilities of this polyphenolic extract. This observed effect cannot be attributed to a single active compound but rather arises from the presence of multiple active compounds that exert additive or synergistic pharmacological effects (Figure 2) [115]. An eight-week feeding experiment was conducted to evaluate the impact of grapevine leaf extract on growth, oxidative enzyme activities, immune response, and the expression of antioxidant genes in zebrafish. Three hundred and sixty zebrafish were provided and fed varying levels of leaf extract (0, 0.5, 1, and 2 g kg^−1^). The research revealed that the leaf extract resulted in an enhancement in the serum and mucus activity of crucial enzymes, such as CAT, SOD, and GPx, which are crucial in eradicating excessive free radicals. Along with an increase in antioxidant enzyme activity, a noticeable reduction in the levels of malondialdehyde was observed. In conclusion, the addition of 0.5^−1^ g kg of Vitis vinifera leaf extract polyphenols to the fish feed serves to enhance the antioxidant defense system (Figure 2) [116].

Achievements already made using zebrafish as a research model are of paramount importance for advancing our understanding in various fields, including CVDs, AS, hyperlipidemia, and antioxidant studies. Traditionally, mammalian models, such as rats and mice, have been predominant in food science research, but the complexity of these models and their relatively slow rate of development have often hindered rapid progress in addressing fundamental nutritional questions. However, the emergence of zebrafish as a highly versatile and genetically tractable model has revolutionized our ability to investigate these complex issues. Zebrafish have been used to evaluate the antioxidant effects of various polyphenols found in food. However, it is noteworthy that relatively few studies have used this model specifically to investigate the polyphenols present in two key dietary components, olive oil and wine. Studies conducted with individual polyphenols or in combination can play a crucial role in exploring potential antioxidant mechanisms. Furthermore, the zebrafish model has significant potential for evaluating the benefits of byproducts generated during olive oil and wine production. These by-products, which often contain bioactive compounds, have the potential to be used as dietary supplements. The antioxidant potential of these byproducts could help us to understand their health-promoting properties and develop novel dietary supplements that could enhance overall well-being.

## 4. Conclusions

Zebrafish have emerged as a valuable but underutilized model for exploring the antioxidant effects of olive oil and wine within the context of the MD. This review highlights the potential benefits of these dietary components in mitigating age-related diseases, particularly cardiovascular conditions. Specifically, the genetic resemblance between zebrafish and humans presents a promising avenue for converting research findings into insights into the health benefits of olive oil and wine. The unique advantages of zebrafish, such as their small size, rapid development, and transparent embryos, make them an economical and efficient model for conducting experiments involving liquid foods, such as olive oil and wine. These features provide a unique opportunity to investigate the effects of these compounds in a controlled and tractable manner. However, it is essential to acknowledge that zebrafish may not fully recapitulate the intricacies of human physiology and metabolism in an experimental model. This limitation underscores the need for caution when extrapolating the zebrafish findings to human health outcomes. In the future, it will be crucial to encourage further exploration of zebrafish models in this context. Researchers should extend the utilization of zebrafish to the investigation of the constituents of the MD and their impact on age-related ailments. In conclusion, it is imperative to conduct more comprehensive studies that delve deeper into the molecular and physiological mechanisms at work and to compare the findings of zebrafish with those of other animal models and clinical trials.
